# Production and upregulation of granulocyte chemotactic protein-2/CXCL6 by IL-1*β* and hypoxia in small cell lung cancer

**DOI:** 10.1038/sj.bjc.6603177

**Published:** 2006-05-23

**Authors:** Y M Zhu, S M Bagstaff, P J Woll

**Affiliations:** 1 Department of Clinical Oncology, Division of Genomic Medicine, School of Medicine and Biomedical Sciences, Institute for Cancer Studies, University of Sheffield, Beech Hill Road, Sheffield S10 2RX, UK

**Keywords:** GCP-2, CXCL6, CXCR1, CXCR2, metastasis, lung cancer

## Abstract

Small cell lung cancer (SCLC) is characterised by early and widespread metastasis. However, SCLC cells have so far been found to produce low levels of known pro-angiogenic factors. We speculated that SCLC cells might produce alternative pro-angiogenic factors. Here, we report that a panel of SCLC cell lines constitutively secrete granulocyte chemotactic protein-2 (GCP-2)/CXCL6, a CXC ELR+ chemokine. In contrast, none of the three tested NSCLC cell lines secreted GCP-2. Production of GCP-2 *in vivo* was also confirmed in seven out of nine specimens with SCLC. We demonstrate that expression of GCP-2 is mediated by NF-*κ*B as ALLN, an NF-*κ*B pathway inhibitor, almost completely abolished GCP-2 production in SCLC cell lines. We also demonstrate that GCP-2 can be significantly upregulated by IL-1*β* and hypoxia in SCLC cell lines. This result suggests a role for GCP-2 in promoting tumour progression *in vivo* under unfavourable conditions such as oxygen deprivation. As SCLC cells express both GCP-2 and its receptors CXCR1 and CXCR2, their biological significance in SCLC progression was further studied. We demonstrate that GCP-2 is an autocrine growth factor. Cell proliferation was significantly inhibited by anti-GCP-2 neutralising antibody in two high-GCP-2-producing cell lines. In addition, expression of the proliferation marker PCNA was upregulated by exogenous GCP-2 in two low-GCP-2-producing cell lines. Taken together, these results suggest an important role for GCP-2 as an autocrine mitogen in the growth and metastasis of SCLC.

Lung cancer is the leading cause of cancer-related deaths worldwide. Small cell lung cancer (SCLC), which has a neuroendocrine phenotype, is characterised by early and widespread metastasis. Despite initial chemosensitivity, resistant relapse is common, and the poor prognosis has not improved in the past 20 years. The establishment of distant metastases, a process that is pathognomic of cancer, depends on angiogenesis. Limited data are available on the contribution of different angiogenic factors to SCLC metastasis.

There is increasing evidence that chemotactic cytokines (chemokines) and their receptors play key roles in cancer progression and metastasis (Balkwill, 2004; Zlotnik, 2004). Many angiogenic factors such as vascular endothelial growth factor (VEGF), basic fibroblast growth factor (bFGF), platelet-derived endothelial cell growth factor (PD-ECGF) and CXC chemokines are associated with tumour progression and metastasis of lung cancer (Cox *et al*, 2000). Among CXC chemokines, those with a conserved Glu-Leu-Arg (ELR+) N-terminal motif are pro-angiogenic, whereas ELR− chemokines inhibit angiogenesis (Strieter *et al*, 1995). The ELR+ CXC chemokines include interleukin-8 (IL-8), growth-related oncogenes-*α*, -*β*, and -*γ*, granulocyte chemotactic protein 2 (GCP-2) and epithelial neutrophil-activating protein-78 (ENA-78). The effects of ELR+ CXC chemokines are mediated through binding to CXCR1 and CXCR2 receptors. All the ELR+ CXC chemokines utilise the CXCR2 receptor, but only IL-8 and GCP-2 recognise the CXCR1 receptor, which we found to be the predominant receptor in lung cancer cells (Zhu *et al*, 2004). Overexpression of IL-8 has been detected in non-small cell lung cancer (NSCLC), where its expression correlates with tumorigenic and metastatic potential (Yuan *et al*, 2000; Chen *et al*, 2003). We have previously studied the expression of IL-8 and its receptors in a panel of NSCLC and SCLC cell lines. We found that whereas NSCLC produced high levels of IL-8, SCLC produced low levels of IL-8. However, SCLC expressed higher levels of the IL-8 receptors, CXCR1 and CXCR2 than NSCLC (Zhu *et al*, 2004). The only other known ligand for CXCR1 receptor is GCP-2. This led us to examine whether SCLC produces GCP-2.

Granulocyte chemotactic protein-2 was cloned as a neutrophil chemoattractant (Proost *et al*, 1993). Like other CXC ELR+ chemokines, GCP-2 was demonstrated to promote tumour growth through its angiogenic effects in animal models (Strieter *et al*, 1995; Van Coillie *et al*, 2001). Recently, Gijsbers *et al* (2005) reported that endothelial cells within gastrointestinal tumours produced GCP-2 that was mitogenic to the endothelial cells and motogenic to the endothelial cells and neutrophils. These results confirmed that GCP-2 could play an important role in human tumour angiogenesis. Here, we show that GCP-2 is constitutively secreted in a panel of SCLC cell lines and in clinical SCLC specimens but not in NSCLC cell lines. We have investigated the regulation of GCP-2 production and its biological functions in SCLC.

## MATERIALS AND METHODS

### Lung cancer cell lines and reagents

The SCLC cell lines used were GLC-19, H69, H345, H711 and Lu165. The NSCLC cell lines used were A549, H460 and MOR/P. All cell lines were cultured in RPMI 1640 (BioWhittaker, Verviers, Belgium) and 10% heat-inactivated FBS (QB perbio, Tattenhall, Cheshire, UK) in humidified 5% CO_2_, 95% air at 37°C. For hypoxic experiments, the cells were incubated in a humidified Heto multigas incubator in 0.5% O_2_, 5% CO_2_ and 95% N_2_. ALLN (Calpain Inhibitor; MG101) was purchased from Calbiochem (Nottingham, UK). Mouse anti-human GCP-2, CXCR1 and CXCR2, and rGCP-2 were purchased from R&D systems (Abingdon, UK). The rabbit anti-human NF-*κ*B (p65) antibody or rabbit anti-human phosphorylated NF-*κ*B (pp65) antibody were purchased from Cell Signaling Technology (Beverly, MA, USA).

### Reverse transcription-polymerase chain reaction (RT–PCR)

Total RNA was isolated by using the RNeasy mini kit (Qiagen, West Sussex, UK) following the manufacturer's protocol. The expression of mRNA for GCP-2 and GAPDH was determined by RT–PCR as described previously (Zhu *et al*, 2003). The primers for GCP-2 are as follows: sense, 5′-CGC TGG TCC TGT CTC TGC T-3′; antisense, 5′-GTT TTT CTT GTT TCC ACT GTC C-3′. The primers for GAPDH are as follows: sense, 5′-CCA CCC ATG GCA AAT TCC ATG GCA-3′; antisense, 5′-TCT AGA CGG CAG GTC AGG TCC ACC-3′. Amplification was carried out in a Biometra thermal cycler after an initial denaturation at 94°C for 3 min. This was followed by 35 cycles of PCR using the following temperature and time profile: denaturation at 94°C for 40 s, primer annealing at 58°C for 40 s, primer extension at 72°C for 1 min, and a final extension of 72°C for 6 min. The PCR products (235 bp for GCP-2 and 593 bp for GAPDH) were visualized by electrophoresis on a 1.5% agarose gel in 0.5 × TBE buffer (44.5 mM Tris borate, 1 mM EDTA, pH 8.3) after staining with 0.5 *μ*g ml^−1^ ethidium bromide.

### Enzyme-linked immunosorbent assay (ELISA)

Granulocyte chemotactic protein-2 concentrations in the culture media were determined by ELISA (R&D systems, Abingdon, UK) as previously described for IL-8 (Zhu *et al*, 2003; Zhu *et al*, 2004). The concentration of GCP-2 was determined by measuring the optical density (OD) at 450 nm in a Dynatech MR5000 microplate reader.

### Flow cytometry analysis of cell surface expression of CXCR1 and CXCR2

The cells were washed twice with phosphate-buffered saline (PBS) and then suspended in 100 *μ*l of FACS buffer (2% bovine serum albumin, 2% normal rabbit serum in PBS), and then 2 *μ*g of mouse anti-human monoclonal anti-CXCR1 or anti-CXCR2 antibody or IgG control antibody (R&D systems) were added and incubated for 40 min on ice. After washing twice with PBS, the cells were suspended in 100 *μ*l of FACS buffer plus 1 : 20 diluted fluorescein isothiocyanate (FITC)-conjugated rabbit anti-mouse IgG (DakoCytomation, Ely, UK) and incubated for 30 min on ice. The cells were washed twice with PBS and fixed in 100 *μ*l of FACS buffer containing 1% paraformaldehyde. The cells were analysed on a FACSort flow cytometer (Becton Dickinson, San Jose, CA, USA).

### Western blotting and immunofluorescences

For NF-*κ*B p65 and pp65 detection, nuclear and cytoplasmic extracts were prepared as follows: cells were first lysed in 10 mM Tris-HCl (pH 7.9), 60 mM KCl, 1 mM EDTA, 1 mM DTT, 1 mM Pefabloc, 50 *μ*g ml^−1^ antipain, 100 *μ*g ml^−1^ chymostatin, 1 *μ*g ml^−1^ leupeptin, 1 *μ*g ml^−1^ pepstatin, 40 *μ*g ml^−1^ bestatin, 3 *μ*g ml^−1^ E-64 and 0.1% NP-40. The cytoplasmic fraction was removed and the pellet resuspended in 20 mM Tris-HCl (pH 8.0), 400 mM NaCl, 1.5 mM MgCl, 1.5 mM EDTA, 1 mM DTT, 25% glycerol, 1 mM Pefabloc, 50 *μ*g ml^−1^ antipain, 100 *μ*g ml^−1^ chymostatin, 1 *μ*g ml^−1^ leupeptin, 1 *μ*g ml^−1^, pepstatin, 40 *μ*g ml^−1^ bestatin and 3 *μ*g ml^−1^ E-64. The nuclear fraction was removed. For GCP-2 detection, cells were lysed in Cellytic™ Mammalian cell lysis extraction buffer (Sigma, Poole, Dorset, England). The protein concentration was determined by the Bradford assay. A measure of 20 *μ*g of protein was used per lane. The detailed procedure from gel eletrophoresis to detection was described previously (Zhu *et al*, 2003). Expression of NF-*κ*B p65 was also detected by immunofluorescence as described by Zhu *et al* (2003). Briefly, SCLC cells were fixed in 4% paraformaldehyde in PBS, permeabilized with 0.2% Triton X-100. Each slide was incubated with blocking solution (1.5% goat serum in PBS), then with diluted rabbit anti-human NF-*κ*B (1 : 200 dilution) antibody, and finally with 1 : 160 diluted FITC-conjugated goat anti-rabbit secondary antibody. The slides were then mounted onto slides with Vectashield mounting medium (Vector Laboratories, Inc., Burlingam, CA, USA). Fluorescence was observed and photographed using a Zeiss microscope.

### MTT assay

Cell proliferation was measured by 3-(4,5-dimethylthiazol-2-yl)-2,5-diphenyltetrazolium bromide (MTT) assay (Sigma, Dorset, England). A total of 5 × 10^3^ cells were seeded into 96-well flat-bottomed plates in triplicate in 100 *μ*l RPMI containing 0.5% FCS. After overnight culture, cells were treated with various concentrations of monoclonal anti-GCP-2 antibody or control antibody mouse IgG for 24 and 48 h. A volume of 10 *μ*l of MTT (5 mg ml^−1^) was added to each well 4 h before the end of experiments. MTT solvent (100 *μ*l of 0.1N Hcl in anhydrous isopropanol) was added and absorbance of the converted dye was measured at a wavelength of 570 nm. The background was also measured at 690 nm.

### Statistical analysis

All results are expressed as mean±s.e.m. The unpaired Student's *t*-test was used to evaluate the significance of differences between groups, accepting *P*<0.05 as the level of significance.

## RESULTS

### Release of granulocyte chemotactic protein-2 from small cell lung cancer but not from non-small cell lung cancer

**Figure 1:**
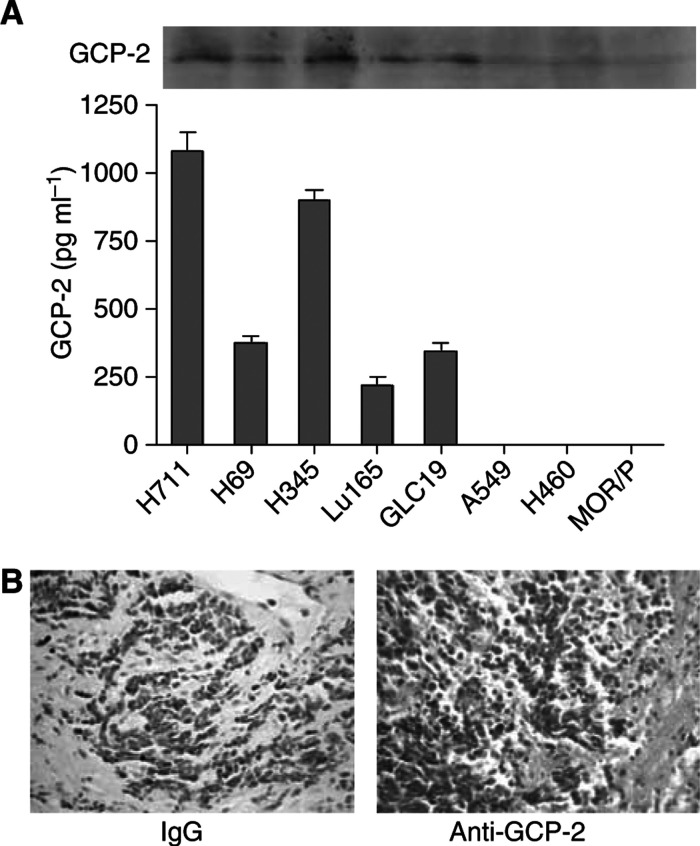
Expression and release of GCP-2 from SCLC cells but not from NSCLC cells. (**A**) GCP-2 protein was measured by ELISA in 24 h-conditioned serum-free medium of a panel of SCLC and NSCLC cell lines. Constitutive release of GCP-2 was found in all the five SCLC cell lines but in none of the three NSCLC cell lines. GCP-2 was also detected in cell lysates from SCLC but not from NSCLC cell lines by Western Blotting. Each bar is the mean±s.e.m. of three determinations from three indepedent experiments. (**B**) Production of GCP-2 *in vivo* was confirmed by immunohistochemistry on biopsy samples in seven out of nine patients (78%).

Granulocyte chemotactic protein-2 protein was measured in conditioned serum-free medium of a panel of SCLC and NSCLC cell lines by ELISA. Constitutive release of GCP-2 was found in all five SCLC cell lines but in none of the three NSCLC cell lines (Figure 1A). After 24 h, the concentration of GCP-2 in the SCLC-conditioned medium was 1080±72 pg ml^−1^/2 × 10^6^ cells in H711, 907±40 pg ml^−1^/2 × 10^6^ cells in H345, 377±24 pg ml^−1^/2 × 10^6^ cells in H69, 346±31 pg ml^−1^/2 × 10^6^ cells in GLC-19 and 216±30 pg ml^−1^/2 × 10^6^ cells in Lu165. GCP-2 was also detected in cell lysates by Western blotting (Figure 1A) although low levels were found in cell lysates by ELISA (data not shown), suggesting that GCP-2 is released from cells soon after synthesis. The production of GCP-2 *in vivo* was confirmed by immunohistochemistry on biopsy samples from nine SCLC patients (Figure 1B). GCP-2 was positive in seven out of nine patients (78%).

### Constitutive expression of granulocyte chemotactic protein-2 is abolished by targeting NF-*κ*B

**Figure 2:**
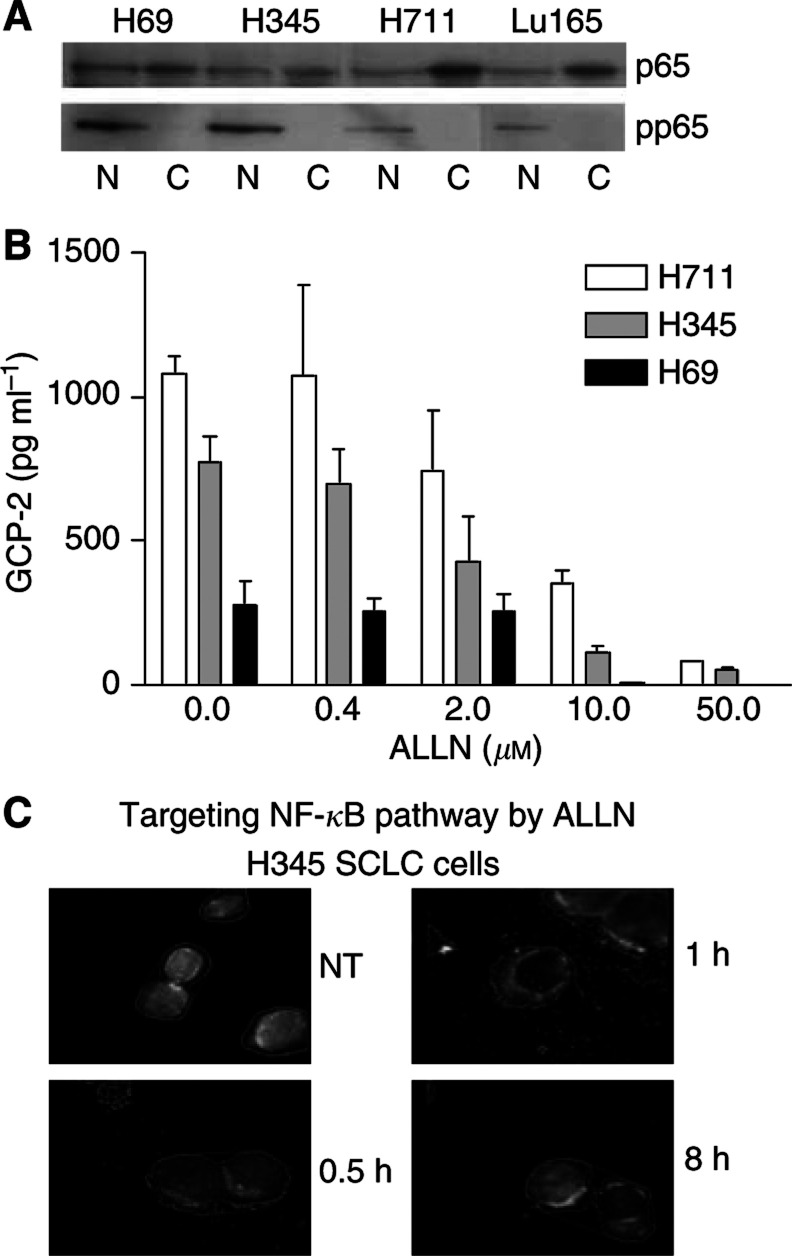
Constitutive expression of GCP-2 in SCLC cells is mediated by NF-*κ*B. (**A**) Expression of NF-*κ*B p65 was detected by Western blotting in both cytoplasmic (C) and nuclear (N) fractions in SCLC cell lines. In contrast, phosphorylated p65 (pp65) was only detected in nuclear fractions in those cells. (**B**) ALLN significantly reduced GCP-2 release, measured by ELISA, in a dose-dependent fashion in H711, H345 and H69 cells. GCP-2 was reduced at least 80% when cells were treated at a final concentration of 10 and 50 *μ*M of ALLN in all SCLC cell lines tested. Each bar is the mean±s.e.m. of three determinations from two independent experiments. (**C**) Nuclear p65, detected by immunofluorescence, was reduced following treatment with ALLN at a final concentration of 10 *μ*M in H345 cells.

The 5′ flanking region of GCP-2 has been cloned and binding sites for three major transcription factors identified there: nuclear factor (NF)-*κ*B, NF-IL-6 and activating protein (AP)-2 (Rovai *et al*, 1997). We investigated the role of NF-*κ*B in regulating GCP-2 activity in SCLC cell lines. Using antibodies specific for total NF-*κ*B p65 and phosphorylated p65, we studied the cellular distribution of phosphorylated and unphosphorylated p65 (Figure 2A). p65 was found in both the nuclear and cytoplasmic cell fractions but phosphorylated p65 was located only in the nucleus. To test whether NF-*κ*B regulated GCP-2 expression, the NF-*κ*B signal pathway was targeted in two high-GCP-2-producing cell lines: H711 and H345. ALLN, which prevents I*κ*B degradation and hence inhibits nuclear translocation of NF-*κ*B (Figure 2C), significantly reduced GCP-2 release in a dose-dependent fashion in both H711 and H345 cells (Figure 2B). GCP-2 was reduced to 355±44 pg ml^−1^ (67% reduction, *P*<0.01) at a final concentration of 10 *μ*M of ALLN and further reduced to 83±4 pg ml^−1^ (92% reduction, *P*<0.001) at 50 *μ*M of ALLN from 1078±26 pg ml^−1^ at control in H711. GCP-2 was reduced to 71±8 pg ml^−1^ (91% reduction, *P*<0.001) at 10 *μ*M of ALLN and further reduced to 41±3 pg ml^−1^ (95% reduction, *P*<0.001) at 50 *μ*M of ALLN from 815±25 pg ml^−1^ at control in H345. Granulocyte chemotactic protein-2 was also reduced to less than 11±1 pg ml^−1^ at 10 *μ*M of ALLN from 277±34 pg ml^−1^ in H69 (96% reduction, *P*<0.001).

### Regulation of granulocyte chemotactic protein-2 secretion by IL-1*β*

**Figure 3:**
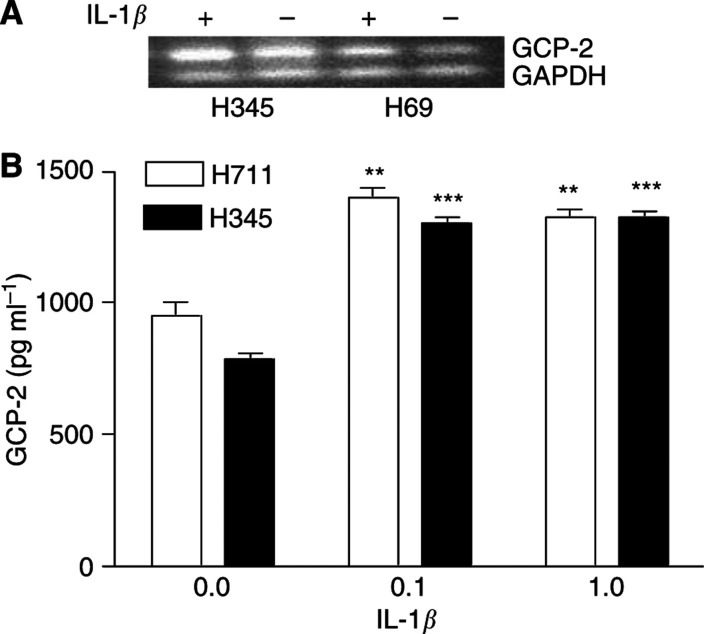
Upregulation of GCP-2 secretion by IL-1*β*. (**A**) IL-1*β* upregulated GCP-2 mRNA detected by RT–PCR in both H711 and H345 cell lines. (**B**) GCP-2 release was upregulated at 0.1 ng ml^−1^ in both H345 (84% increase, *P*<0.01) and H711 (53% increase, *P*<0.01) cell lines, and at 1 ng ml^−1^ after H345 and H711 cells were treated by rIL-1*β* for 24 h. Each bar is the mean±s.e.m. of three determinations.

IL-1*β* has been shown to be the predominant inducer of GCP-2 in non-tumour cells such as mesenchymal cells (Wuyts *et al*, 2003) and endometrial stromal cells (Mine *et al*, 2003). To assess whether IL-1*β* induces GCP-2 in SCLC cell lines, the high-GCP-2-producing H345 and H711 cells were treated by rIL-1*β* at a final concentration of 0.1 or 1 ng ml^−1^ for 24 h (Figure 3A). A significant increase in GCP-2 release was found at 0.1 ng ml^−1^ in both H345 (84% increase, *P*<0.01) and H711 (53% increase, *P*<0.01) cell lines. No further increase of GCP-2 was found when IL-1*β* was increased to 1 ng ml^−1^ (Figure 3A). As shown by RT–PCR, IL-1*β* upregulated GCP-2 mRNA in both cell lines, indicating that the upregulation is mediated by increased transcription of GCP-2 (Figure 3B).

### Upregulation of GCP-2 by hypoxia

**Figure 4:**
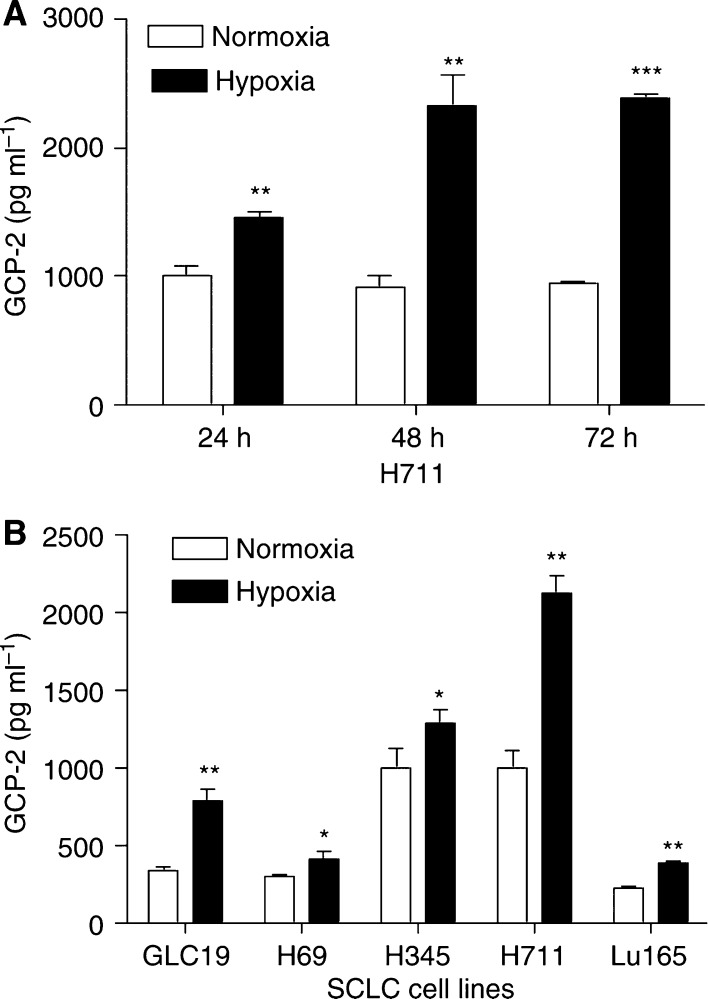
Upregulation of GCP-2 by hypoxia. (**A**) H711 cells were cultured under normoxia and hypoxia (0.5% O_2_) for 24, 48 and 72 h. Granulocyte chemotactic protein-2 measured by ELISA was significantly increased by 46% (*P*<0.01) at 24 h, 152% (*P*<0.01) at 48 h and 153% (*P*<0.001) at 72 h. (**B**) The effects of 48 h hypoxia on GCP-2 production in a panel of SCLC cell lines. Granulocyte chemotactic protein-2 production measured by ELISA was significantly upregulated (range from 34% in H69 to 129% increase in GLC19) in all tested SCLC cell lines. Each bar is the mean±s.e.m. of three determinations from two independent experiments.

To test whether hypoxia upregulates GCP-2, H711 cells were cultured under normoxia and hypoxia (0.5% O_2_) for 24, 48 and 72 h (Figure 4A). GCP-2 was increased from between 1000±80 (24 h) and 940±20 (72 h) pg ml^−1^ under normoxia to 1460±40 pg ml^−1^ under hypoxia (46% increase, *P*<0.01) at 24 h, to 2320±240 pg ml^−1^ (152% increase, *P*<0.01) at 48 h and to 2380±40 pg ml^−1^ (153% increase, *P*<0.001) at 72 h. We further studied the effects of 48 h hypoxia on GCP-2 production in a panel of SCLC cell lines. Granulocyte chemotactic protein-2 production was significantly upregulated (range from 34% in H69 to 129% increase in GLC19) in all tested SCLC cell lines (Figure 4B).

### Granulocyte chemotactic protein-2 is an autocrine mitogen for small cell lung cancer cells

**Figure 5:**
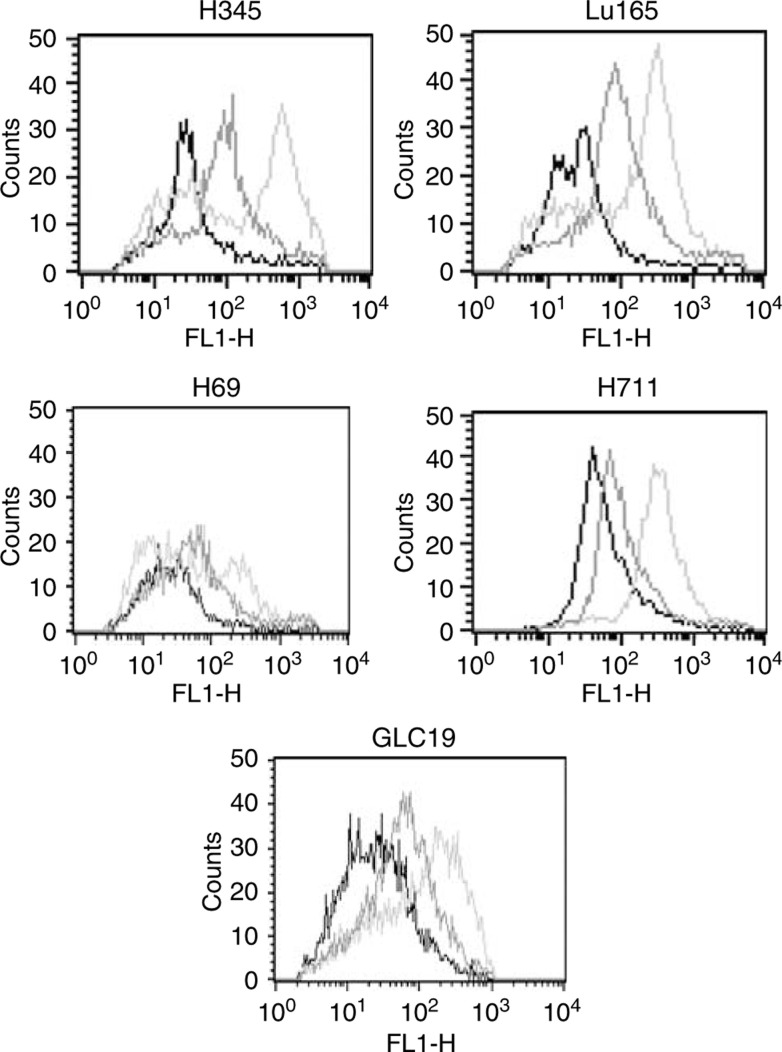
Expression of CXCR1 and CXCR2 on SCLC cells. Expression of the CXCR1 and CXCR2 was detected by FACS in a panel of SCLC cell lines. All the SCLC cell lines express functional GCP-2 receptors. Expression of CXCR1 (right histogram) were higher than CXCR2 (middle histogram) in all SCLC cell lines.

**Figure 6:**
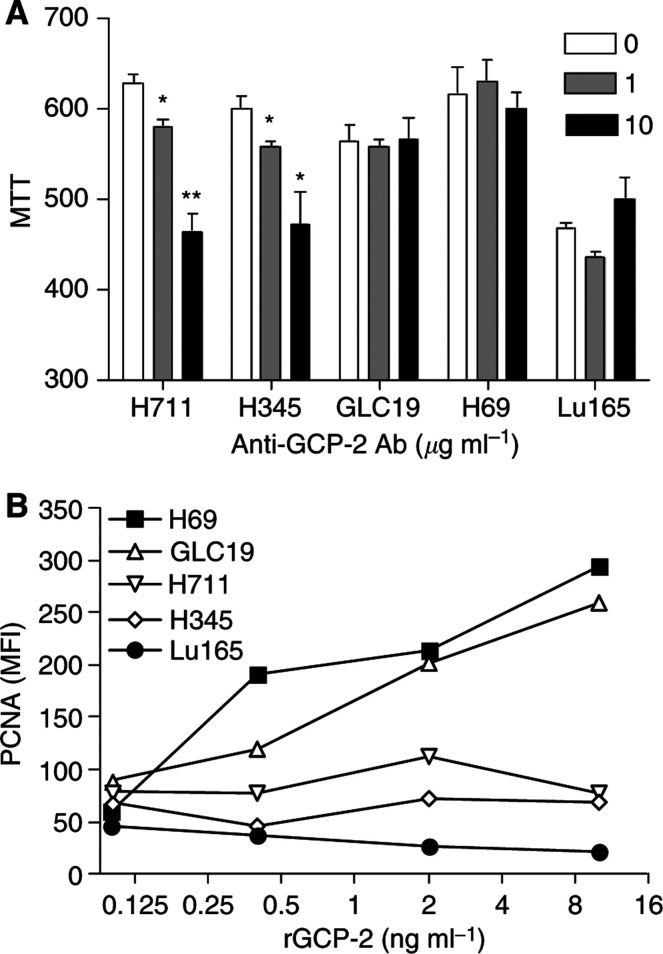
Granulocyte chemotactic protein-2 is an autocrine mitogen for SCLC cells. (**A**) After SCLC cells were treated with neutralising anti-GCP-2 antibody for 48 h, cell proliferation was significantly inhibited by 26% (*P*<0.01) in H711 and by 21% (*P*<0.05) in H345, but not affected in H69, GLC19 and Lu165. Each bar is the mean±s.e.m. of three determinations from two independent experiments. (**B**) The expression of PCNA was measured by flow cytometry following the treatment of exogenous GCP-2. The results are expressed as the mean fluorescent intensity. rGCP-2 (0.1–10 ng ml^−1^) upregulated the expression of PCNA in dose-dependent manner in two low-GCP-2-producing cell lines H69 and GLC19. In contrast, expression of PCNA was not increased after rGCP-2 treatment in high-GCP-2-producing cell lines H711 and H345, or in Lu165.

Expression of the GCP-2 receptors CXCR1 and CXCR2 was studied by FACS. We found that all the SCLC cell lines express functional GCP-2 receptors (Figure 5). As SCLC cells express both GCP-2 and its receptors, we speculated that it could act as an autocrine growth factor for these cells. To test this, SCLC cells were treated with neutralising anti-GCP-2 antibody for 48 h. As measured by MTT assay, cell proliferation was significantly inhibited by 26% (*P*<0.01) in H711 and by 21% (*P*<0.05) in H345, but cell lines GLC-19, H69 and Lu165 were not significantly affected (Figure 6A). We also studied the effects of exogenous GCP-2 on cell proliferation by examining the expression of the proliferation marker, proliferating cell nuclear antigen (PCNA) by flow cytometry with mouse anti-human PCNA monoclonal antibody and control mouse IgG2a antibody. The results are expressed as the mean fluorescent intensity. Recombinant GCP-2 (0.1–10 ng ml^−1^) upregulated the expression of PCNA in a dose-dependent fashion in two low-GCP-2-producing cell lines H69 and GLC19. Expression of PCNA increased five-fold in H69 and three-fold in GLC-19 cells when the cells were treated with rGCP-2 at a final concentration of 10 ng ml^−1^ (Figure 6B). In contrast, expression of PCNA was no significantly increased after rGCP-2 treatment in the two high-GCP-2-producing cell lines, H711 and H345, or in Lu165.

## DISCUSSION

Granulocyte chemotactic protein-2 was originally isolated from conditioned medium of osteosarcoma cells (Proost *et al*, 1993). Subsequent studies have shown that GCP-2 was produced in tumour cell lines such as MG-63, HeLa, and Bowes. It has been shown to have mitogenic and angiogenic properties in different models (Strieter *et al*, 1995; Van Coillie *et al*, 2001). Like other ELR+ CXC chemokines, GCP-2 was chemotactic to endothelial cells *in vitro* and promoted rat cornea neovascularization *in vivo* (Strieter *et al*, 1995). Van Coillie *et al* (2001) showed that tumour-derived GCP-2 increased angiogenesis through activation of tumour-associated neutrophils and intratumoral expression of gelatinase B. GCP-2 binds to both CXCR1 and CXCR2 with similar high affinity. Both receptors are G protein-coupled seven-transmembrane domain receptors, but different biological functions may be mediated by the different receptors. CXCR1 is believed to mediate the chemotactic activity, whereas CXCR2 mediates the angiogenic activity of CXC ELR+ chemokines such as IL-8 (Heidemann *et al*, 2003; Ramjeesingh *et al*, 2003; Bates *et al*, 2004).

Angiogenesis and metastasis are essential to tumour development. Angiogenic factors have been extensively described in NSCLC but not in SCLC. Expression of VEGF was detected in 43 of 54 (81%) patients with SCLC, but was not associated with survival (Dowell *et al*, 2004) although increased expression of VEGF in SCLC was associated with poorer prognosis in another indepedent study (Fontanini *et al*, 2002). To date SCLC cells have been found to produce lower levels than NSCLC of pro-angiogenic factors such as VEGF and IL-8 (Yatsunami *et al*, 1997). This suggests that SCLC might utilise different angiogenic factors than NSCLC. In this study, we report that SCLC cells secrete GCP-2, whereas NSCLC cells do not, as shown by ELISA of conditioned media in cell culture, Western blotting of cell lysates and immunohistochemistry of tumour specimens. This finding demonstrates that SCLC cells secrete an additional pro-angiogenic factor to NSCLC. We therefore further studied the regulation and function of GCP-2 in SCLC.

We investigated the role of NF-*κ*B (p65), a crucial transcription factor that mediates chemokine induction, in GCP-2 secretion by exposing cells to ALLN, which stabilises I*κ*B. ALLN almost completely abolished GCP-2 secretion in H345 and H711 cells, suggesting that NF-*κ*B is a major regulator of GCP-2 expression in SCLC. We further tested whether IL-1*β* upregulated GCP-2 in SCLC cells. Both mRNA and protein of GCP-2 were significantly increased in two SCLC cell line tested. This result suggests that upregulation of GCP-2 by IL-1*β* is initiated at the transcriptional level. IL-1*β* has been found to induce NF-*κ*B activation in cancer cells such as leukaemic cell line OCIM2 (Estrov *et al*, 1999). Therefore, upregulation of GCP-2 by IL-1*β* may be mediated by NF-*κ*B induction.

Granulocyte chemotactic protein-2 is one of the CXC ELR+ chemokines with pro-angiogenic properties. GCP-2 has previously been shown to act as an angiogenic factor (Strieter *et al*, 1995). Many angiogenic factors are induced when tumour cells are exposed to unfavourable conditions such as hypoxia. We therefore investigated the role of hypoxia on GCP-2 production in SCLC cells. We found that GCP-2 was significantly increased in all SCLC cell lines after 48 h in 0.5% O_2_. Among them, GCP-2 production was increased by over 120% in H711 and GLC19 cell lines. The underlying mechanism of upregulation of GCP-2 by hypoxia in SCLC has not yet been defined. We scrutinised the promoter region of GCP-2 (Mine *et al*, 2003), and found a potential hypoxia-inducable factor (HIF) DNA binding site (GCGTG) (Wenger and Gassmann, 1997) present in the hypoxia-response element (HRE) from −430 to −424 within the promoter of GCP-2. It has been well documented that HIF is rapidly stabilised upon exposure to hypoxia (Wenger, 2000). Therefore, HIF may mediate GCP-2 upregulation by hypoxia in SCLC cells. In addition to HIF, NF-*κ*B may play a role in the upregulation of GCP-2 by hypoxia. Hypoxia can upregulate genes without an HRE in their promoters and such hypoxia-induced gene induction was NF-*κ*B-dependent (Karashima *et al*, 2003). The mechanism of GCP-2 upregulation by hypoxia merits further study.

As SCLC cells expressed CXCR1 and CXCR2 on the cell membrane and secrete GCP-2, we further investigated the biological functions of GCP-2 in SCLC in this study. Expression of PCNA, a marker of cell proliferation, was significantly increased after SCLC cells were treated with rGCP-2 in at least two low GCP-2-producing cell lines GLC19 and H69. Cell proliferation was inhibited after the cells were treated with neutralising anti-GCP-2 antibody in two high-GCP-2-producing cell lines (H711 and H345), but not in three low-GCP-2-producing cell lines GLC19, H69 and Lu165. These results suggest that GCP-2 may act as an autocrine growth factor in SCLC cells. Interleukin-8 (IL-8), which shares the CXCR1 and CXCR2 receptors with GCP-2, was also found to be mitogenic to SCLC (Zhu *et al*, 2004). The signal transduction pathways mediating the mitogenic function of IL-8 are complex, include extracellular signal-regulated kinase (ERK) of mitogen-activated protein kinase (MAPK) pathway, NF-*κ*B, and epidermal growth factor receptor (EGFR) pathway by cross-talk (Zhu and Woll, 2005). As the GCP-2 binds to the same receptors, we speculate that the same pathways will be utilised.

In summary, we showed that SCLC cells constitutively secrete GCP-2 chemokine but NSCLC cells do not. GCP-2 production can be significantly upregulated by IL-1*β* and hypoxia. Granulocyte chemotactic protein-2 production is mediated by NF-*κ*B. In addition to its angiogenic property previously shown in other studies, we demonstrated that GCP-2 can act as an autocrine growth factor to most SCLC cells. Thus, GCP-2 could be critical to the tumour development in SCLC. These results suggest that targeting GCP-2 may inhibit tumour growth in SCLC.
